# Unveiling the Nexus: Cellular Metabolomics Unravels the Impact of Estrogen on Nicotinamide Metabolism in Mitigating Rheumatoid Arthritis Pathogenesis

**DOI:** 10.3390/metabo14040214

**Published:** 2024-04-11

**Authors:** Swati Malik, Debolina Chakraborty, Prachi Agnihotri, Vijay Kumar, Sagarika Biswas

**Affiliations:** 1 Department of Integrative and Functional Biology, CSIR—Institute of Genomics and Integrative Biology, Mall Road, Delhi 110007, India; swati.malik@igib.res.in (S.M.); debolina@igib.res.in (D.C.); prachi.igib22a@acsir.res.in (P.A.); 2 AcSIR—Academy of Scientific and Innovative Research, Ghaziabad 201002, India; 3 Department of Orthopaedics, AIIMS—All India Institute of Medical Sciences, Ansari Nagar, New Delhi 110029, India; vkgene@aiims.edu

**Keywords:** metabolomics, estrogen, 1-methylnicotinamide, nicotinate and nicotinamide metabolism, synovial fibroblast, rheumatoid arthritis

## Abstract

Rheumatoid arthritis (RA) is a metabolic joint disorder influenced by hormonal regulation, notably estrogen, which plays a cytoprotective role against inflammation. While estrogen’s impact on RA pathogenesis has been studied, the altered metabolite expression under estrogen’s influence remains unexplored. This study investigated the changes in the metabolome of synovial fibroblasts isolated from RA patients under 17β-estradiol (E2) using the liquid chromatography with tandem mass spectrometry (LC-MS/MS) approach followed by multivariate and biological pathway analysis along with in vitro validation. Results identified 3624 *m*/*z*, among which eight metabolites were significant (*p* < 0.05). Nicotinate and nicotinamide metabolism was found to be highly correlated with the treatment of E2, with metabolites NAD^+^ and 1-methynicotinamide (1-MNA) upregulated by E2 induction in RA-FLS. PharmMapper analysis identified potential gene targets of 1-MNA, which were further matched with RA gene targets, and thus, STAT1, MAPK14, MMP3, and MMP9 were concluded to be the common targets. E2 treatment affected the expression of these gene targets and ameliorated the development of oxidative stress associated with RA inflammation, which can be attributed to increased concentration of 1-MNA. Thus, an LC-MS/MS-based metabolomics study revealed the prominent role of estrogen in preventing inflammatory progression in RA by altering metabolite concentration, which can support its therapeutic capacity in remitting RA.

## 1. Introduction

Rheumatoid arthritis (RA) is a chronic autoimmune disorder with altered metabolism that correlates with progressive inflammatory conditions [1]. Affecting the synovial joints in the body, the condition is characterized by joint pain, swelling, and stiffness, often associated with disability and sometimes premature death [2]. Impacting a substantial population in the world, RA remains a major public health challenge [3]. Although the number of therapeutic resources available at the disposal of RA patients has increased tremendously in the form of providing non-steroidal anti-inflammatory drugs (NSAIDs), and treatment with disease-modifying anti-rheumatic drug (DMARD) therapy, there is still a lack of efficacy in these treatment options for high-risk individuals [4,5]. Studying the pathophysiology of RA and identifying the factors associated with disease activity will help develop therapeutic strategies for sustained remission [6].

Hormones are also involved in various aspects of RA progression, and a considerable level of crosstalk exists between hormonal perturbations and RA metabolism [7]. Epidemiological evidence of RA indicates sexual disparity in terms of its greater prevalence in females than males [8]. This has been indicated to be because of the prominent role of estrogen in the development of RA [9]. The risk of development of RA has been reported in post-menopausal women, which suggests an association of estrogen deficiency with peak incidence of RA [10]. Apart from RA, estrogen has also been observed to play an essential role in the pathogenesis of other autoimmune dysregulations, such as multiple sclerosis [11]. The prominent evidence of estrogenic regulation of RA development comes from studies that found that, in women suffering from RA, the disease activity decreases with the increase in the female hormone estrogen [12,13]. In animal models of collagen-induced arthritis (CIA), extensive studies have shown that estrogen deficiency in the form of ovariectomy (OVX) aggravates the severity of arthritis and estrogen induction mediates the suppression of disease development [14,15]. Estrogen modulates the inflammatory progression in RA at different levels. It was found to decrease the enhanced production of pro-inflammatory cytokines like IL (Interleukin)-1β and IL-6 in TNF-α-stimulated arthritic fibroblasts [16], and to downregulate the expression of transforming growth factor β-activated kinase-1 (TAK1), which activates proinflammatory cytokine signaling pathways downstream [17]. Furthermore, estrogen also regulates the innate immune response by increasing IgG-Fc sialylation in post-menopausal women with RA [18]. We recently reviewed the effect of estrogen on the differential proteome of RA, along with in silico analysis that showed various signaling pathways and related proteins involved in the inflammatory progression of RA were the targets of estrogenic therapeutic efficacy [19].

Regarding modulating therapeutic strategies in RA, recent studies have identified the significance of metabolites and the metabolic pathways [20] as attractive targets of treatment. The inflammatory and immune response in RA entails an altered level of intermediate metabolites involved in different metabolic pathways [21], which are responsible for significant cellular adaptations, such as cell proliferation [22], the hypoxic microenvironment [23], and inflammatory cell infiltration [24]. Different studies have also identified the role of estrogen in closely regulating metabolic intermediates in estrogen-treated disease conditions [25], which can be further utilized to explore its therapeutic potential. However, minimal information is available on the effect of estrogen in regulating these intermediate metabolic alterations in RA. 

Metabolomics is a new area of study that focuses on analyzing low molecular weight (molecular weight < 1500 Da) molecules called metabolites in the biological samples [26]. These metabolites could be a part of biological processes or derived from outside sources like food and drugs [27,28]. Metabolomics, an advanced technique, is widely applied to identify the biological perturbation and potential biomarkers in RA pathogenesis [29,30]. The altered amino acid metabolism identified by liquid chromatography–mass spectrometry (LC/MS) in CIA mice plasma was associated with muscle mass wasting and protein degradation [31], which occur during the inflammatory progression of RA. Similarly, serum metabolomics in RA patients has also identified distinctive metabolic signatures distinguishing them from other autoimmune disorders, such as primary Sjogren’s syndrome [32]. This signifies the potential of metabolic analysis in studying and identifying the underlying pathogenic mechanism of the disease. Metabolomics analysis has also aided in determining the metabolic alteration that benefits from treatment [33]. The different metabolomics studies, however, have not provided the knowledge about identifying the treatment responses from the site of inflammation involving stromal subsets in the synovial joints. The current study represents a critical analysis in determining the therapeutic efficacy of estrogen by identifying the metabolic alterations caused by its treatment in the main effector cells, synovial fibroblasts, which are present at the site of inflammation and can provide valuable information for clinical applications of estrogen in RA. 

In this study, liquid chromatography with tandem mass spectrometry (LC-MS/MS) was used to examine alterations in metabolites in RA fibroblast-like synoviocytes (RA-FLS), after treatment with exogenous 17β-estradiol (E2). Our aim was to investigate the differential metabolites that are affected by the E2 treatment during RA conditions. The 18-carbon steroid E2 is predominantly the most active form of estrogen [34]. Thus, it was utilized for estrogenic treatment in cells. The LC-MS/MS-based metabolomic approach used in this study offers advantages by combining LC with MS, thus helping in metabolic separation before detection, and also improving sensitivity and reducing sample complexity [35]. Using this cutting-edge technology, we observed upregulation of nicotinamide metabolism, which mitigates RA progression after E2 treatment. Altered nicotinamide metabolism via E2 exposure has therapeutic intervention potential in RA conditions. 

## 2. Materials and Methods

### 2.1. Clinical Sample Collection 

Synovial tissue was collected from RA female patients (*n* = 6) who met the revised 2010 American College of Rheumatology (ACR) and European League Against Rheumatism (EULAR) diagnosis criteria from the Department of Rheumatology, All India Institute of Medical Sciences (AIIMS), New Delhi, India. The patients’ clinical demography and healthy control data are provided in Supplementary Table S1. The study protocol received ethical approval from the Council of Scientific and Industrial Research (CSIR)–Institute of Genomics and Integrative Biology, Delhi, India (CSIR-IGIB/IHEC/2017-18 Dt. 8 February 2018) and AIIMS, New Delhi, India (Reg No IEC-237/ 7 May 2021, RP-18/2021). All participants in the study signed the informed consent at enrolment.

### 2.2. Cell Culture and Treatment

FLS were derived from the synovial tissue of RA patients based on the tissue culture method [36]. The cells were isolated by digesting the synovial tissue with collagenase (SIGMA) (0.5 mg/gm of tissue). The tissue suspension was then passed through a cell strainer (100 µ pore size, BD), cultured in a T-75 tissue culture flask in complete DMEM, supplemented with 10% fetal bovine serum (FBS) at 37 °C in 5% CO_2_, and grown to 70–80% confluency for experimental analysis. The RA-FLS were seeded and allowed to adhere, followed by E2 treatment (1 µM) in phenol red-free DMEM for 24 h, and control RA FLS were untreated [37,38]. 

### 2.3. Metabolite Extraction

For metabolite extraction, RA-FLS were seeded in a T-75 cm^2^ cell culture flask. The samples were categorized as RA-FLS untreated (RA control) (*n* = 3) and RA-FLS treated with E2 at a concentration of 1 µM for 24 h (RA + E2 (1 µM)) (*n* = 3). The cells were then trypsinized, and the cell pellet was collected. The metabolites from the cell pellet were extracted by dissolution in chilled 80% methanol, incubated at −20 °C for 1 h followed by centrifugation, and the obtained supernatant was analyzed for differential metabolites by two complementary LC-MS/MS metabolomics methods to allow metabolite separation: hydrophilic interaction LC (HILIC) and reversed-phase LC (RPLC) using C18 columns, each with positive and negative ion modes. The HILIC method was used to separate polar compounds and RPLC for separation of nonpolar analytes [39].

### 2.4. Data Processing

The raw LC-MS (.wiff format) data file was allowed to enter in Peak View (ABSciex). The total ion chromatogram (TIC) normalization was carried out by using a total sum area-based module via Marker View (ABSciex). TIC is a chromatogram formed by totaling intensities of all mass spectral peaks related to the same scan. For appropriate TIC, all the intensities were superimposed. The standardized intensities were then calculated for each sample for different datasets in a spreadsheet. 

The obtained metabolite file of each HILIC and C18 column with both positive and negative ion modes was analyzed against the NIST library “https://chemdata.nist.gov/” (accessed on 25 August 2023) and MS-Dial tool “http://prime.psc.riken.jp/compms/msdial/main.html/” (accessed on 25 August 2023) to annotate the metabolites. The library contained retention time/index (RI), mass-to-charge ratio (*m*/*z*), and chromatographic data, including MS/MS spectral data. Biochemical identification was based on three other criteria, as follows: (1) retention index within a narrow retention time (RT) window of the proposed identification, (2) accurate mass match to the library ±10 ppm, and (3) MS/MS forward and reverse scores between the experimental data and authentic standards. Each metabolite was identified by evaluating corresponding mass and retention time (RT) with a mass precision gap of 10 ppm, window RT ± 2 min, and library score of more than 0.5.

The volcano plot of differential metabolites was generated using Prism 9.0 software (GraphPad, La Jolla, CA, USA). The total annotated metabolites were analyzed by MetaboAnalyst 6.0 to examine the pathway analysis of the annotated metabolites and the significant metabolites. Fold change >1.5 and <0.83 with *p*-value < 0.05 was classified to screen significant differential metabolites. The heat map, Kyoto Encyclopedia of Genes and Genomes (KEGG) pathway, and enrichment analysis were processed with MetaboAnalyst 6.0. 

### 2.5. Target Prediction of Metabolites by PharmMapper Analysis

The potential gene target identification for small molecules is an essential aspect of the drug discovery spectrum. The gene targets of the identified metabolite were analyzed by PharmMapper analysis [40]. The structure of the metabolite in Spatial Data File (SDF) format was retrieved from PubChem (https://www.ncbi.nlm.nih.gov/pccompound/) and was uploaded to the PharmMapper database (https://www.lilab-ecust.cn/pharmmapper/). The obtained gene targets of the metabolite were matched with the gene targets of RA, as retrieved from the DisGeNET database (https://www.disgenet.org/), which has the collection of all the genes associated with the specific disease [41]. Our previous work has published the total genes involved in the progression of RA and retrieved from the database [42]. The matched gene targets of candidate metabolites and RA targets enabled us to recognize the therapeutic potential of metabolites in targeting RA remission. 

The matched gene targets were analyzed to construct a protein–protein interaction (PPI) network using STRING 11.0 software (https://string-db.org) that provides for the functional link between the proteins [43]. The protein interaction network, Gene Ontology (GO) enrichment, KEGG, and Reactome pathway analysis of the common targets were also obtained and visualized using Cytoscape 3.7.0 software (https://cytoscape.org). 

### 2.6. RNA Isolation and qRT-PCR

The RNA isolation was performed by extracting the RA control and E2-treated RA-FLS in Tri-Xtract Reagent (G-biosciences), followed by cDNA synthesis (G-biosciences) preparation as per the manufacturer’s protocol [44]. Quantitative real-time polymer chain reaction (RT-PCR) was performed using HOT FIREPol EvaGreen qPCR Mix Plus (Solis Biodyne) followed by amplification using the Roche Light Cycler^®^ 480 Instrument-II RT-PCR detection system. The fold change in gene expression was measured using the delta threshold cycle (ΔCt), and the fold change was normalized with the housekeeping gene Glyceraldehyde 3-phosphate dehydrogenase (GAPDH). Each reaction was performed in triplicate [44]. The primer sequences of all genes are given in Table S2.

### 2.7. Western Blotting

The cell lysate was prepared from RA control and E2-treated RA-FLS through lysis in Radio-Immunoprecipitation Assay (RIPA) buffer (SIGMA) for Western blotting. Protein concentration was measured using BCA assay (G-biosciences) [45]. The 20 µg protein was resolved on 10% SDS-PAGE gel and then transferred to a nitrocellulose (NC) membrane at 20 V for 50 min using a semi-dry transfer unit (Bio-Rad, Hercules, CA, USA) followed by overnight blocking at 4 °C with 5% bovine serum albumin (BSA) in 1X Tris-buffered saline-tween 20 (TBST) [44]. The membrane was then incubated with primary antibodies STAT1 (9172T, Cell Signaling), Phospho-STAT1 (Tyr701) (9167L, Cell Signaling) (dilution 1:5000) overnight at 4 °C followed by washing and incubation with horseradish peroxidase (HRP)-conjugated anti-mouse secondary antibody (dilution 1:8000) (Jackson, West Grove, PA, USA) for 1 h at room temperature. The band intensity was measured by chemiluminescence (ECL) reagent (Thermo Scientific, Rockford, IL, USA). The band intensity was normalized by the housekeeping protein (β-actin; sc-47778) as a loading control. 

### 2.8. Detection of Total Cellular ROS Production

Excessive ROS production is primarily responsible for generating oxidative stress in the cellular machinery [46]. Cellular ROS was quantitated in RA control and E2-treated RA-FLS using 2′,7′-dichlorodihydrofluorescein diacetate (DCFHDA) (Invitrogen). DCFHDA assesses total ROS containing hydroxyl radicals (^•^OH) and nitrogen dioxide (^•^NO_2_) via its oxidation to fluorescent 2′-7′dichlorofluorescein (DCF) [47]. RA FLS, after respective treatment, were incubated with 10 µM DCFHDA in phenol red-free DMEM at 37 °C with 5% CO_2_ for 30 min. After that, the cells were washed with 1X phosphate buffered saline (PBS) [48]. The fluorescence images were captured using a ZOE fluorescent cell imager (Bio-Rad, Hercules, CA, USA) under an excitation wavelength of 495 nm and an emission wavelength of 529 nm. The brightfield images of the cells within the same scale bar (100 µm) and area were also captured to analyze the number of cells. The fluorescence intensity was quantified by ImageJ analysis software (version 1.41; National Institute of Health (NIH), Bethesda, Maryland, USA) via generation of 8-bit images for intensity quantification, which was normalized to cell count [49].

### 2.9. Statistical Analysis

The results are expressed as mean ± standard deviation (S.D.) The data were analyzed with Student’s *t*-test or the one-way Analysis of variance (ANOVA) test using Prism 9.0 software (GraphPad, La Jolla, CA, USA). *p* < 0.05 (two-tailed) was considered significant.

## 3. Results

### 3.1. Differential Metabolomic Analysis of RA Synovial Fibroblast upon Estradiol Induction

**Figure 1:**
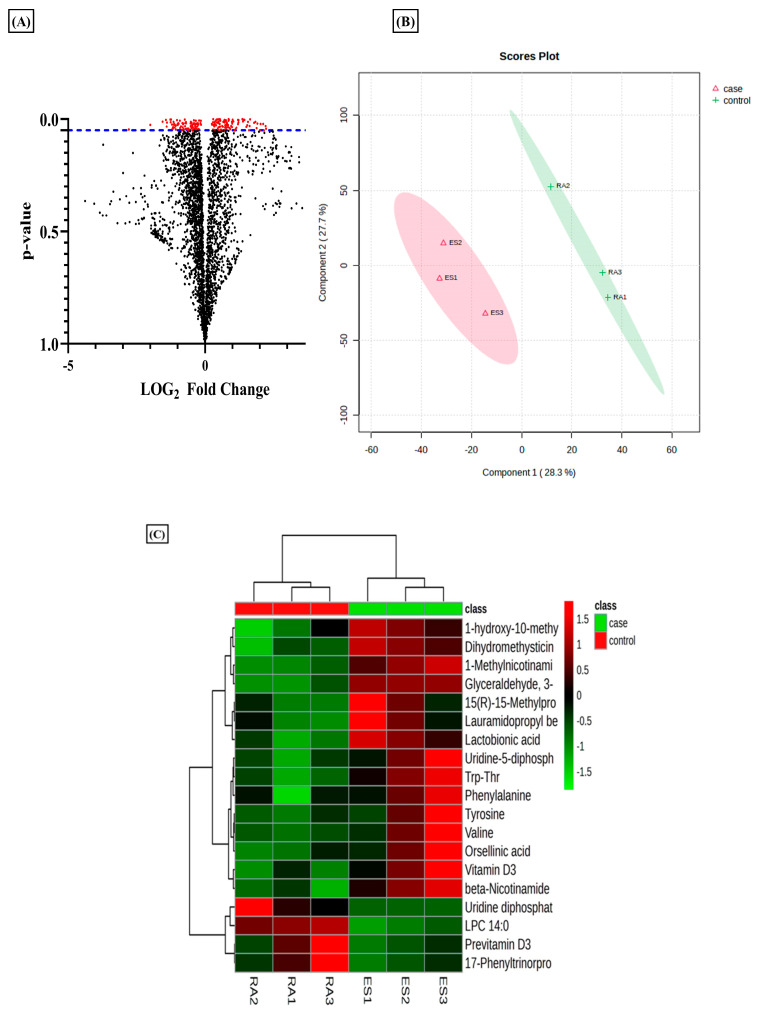
Metabolomic differences between untreated rheumatoid arthritis (RA) synovial fibroblast (FLS) and estradiol (E2) RA-FLS with 1 µM for 24 h. (**A**) Volcano plot of differential metabolites identified; each dot in the plot represents the identified 3624 altered metabolites and significant threshold set at *p*-value < 0.005; the 176 significant metabolites with red dots are distinguished from the rest of metabolites represented by black dots. (**B**) PSL-DA score plot from total cellular metabolites showing differences between untreated RA-FLS and E2-treated RA-FLS. (**C**) Heat map showing the metabolic difference between the two groups represented by color gradient variation from red to green, *n* = 3 per group.

Metabolomics evaluation via a tandem MS/MS experiment has been used to identify the metabolites using their *m*/*z* value searched against the databases [35]. In the current analysis, we used this tool to evaluate the differential metabolites in RA-FLS treated with E2 compared to RA-FLS control. The results revealed that estradiol profoundly impacts metabolic alteration in RA patients. The total identified *m*/*z* from the metabolomic analysis was 3624, which yielded 176 significant *m*/*z* features (*p*-value < 0.05) indicated by red dots in the volcano plot (Figure 1A). Projections to Latent Structures–Discriminant Analysis (PLS-DA) of the total metabolomic profile identified a relative metabolome separation between the RA-FLS control and E2-treated RA-FLS (Figure 1B), depicting a significant separation between the two groups in a case–control study. Heatmap clustering of identified metabolites expressed significant differences between case–control groups, as shown in Figure 1C. It demonstrated a changing pattern of metabolite concentrations between control and treated samples represented by color gradient variations, which shows that E2 impacts the concentration of metabolite in RA-FLS.

### 3.2. Estradiol Promotes Nicotinamide Metabolism in RA Synovial Fibroblasts

**Figure 2:**
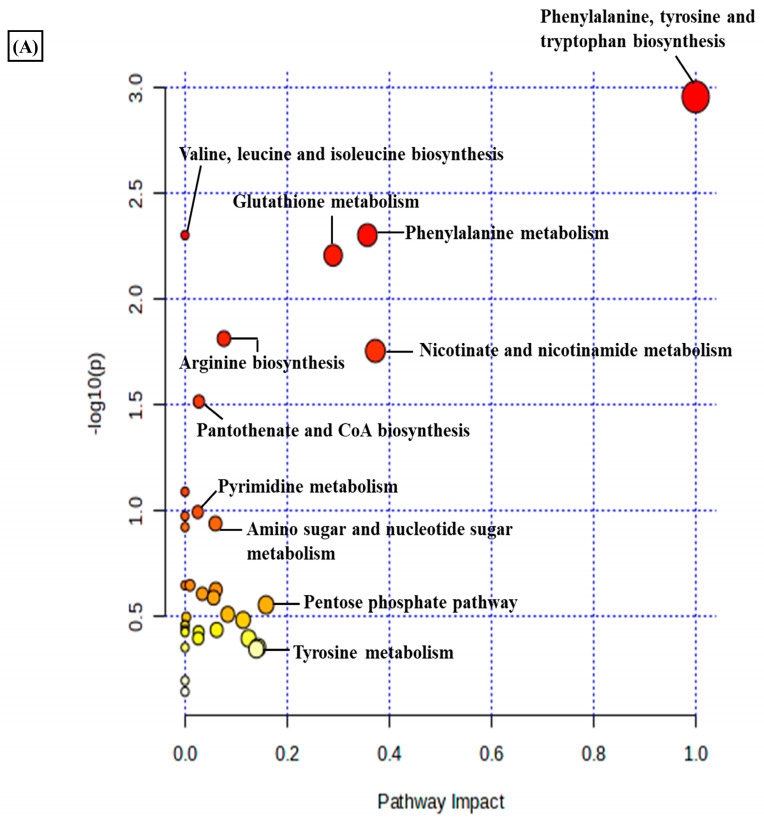

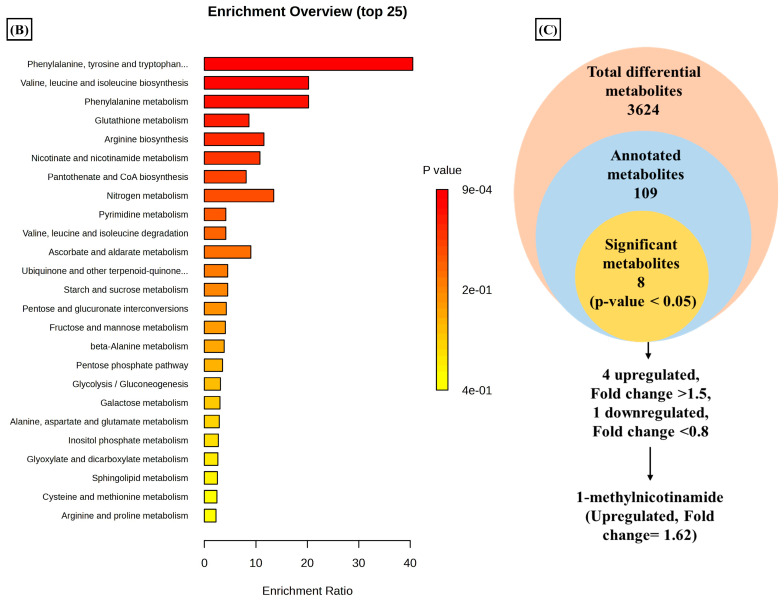
Metabolic pathway analysis using MetaboAnalyst 6.0 (**A**) KEGG pathway analysis of 109 annotated metabolites; the node depicts the *p*-value of the analyzed pathways, and the node radius shows the impact of the pathways. (**B**) Histogram representation of KEGG enrichment analysis of the 109 annotated metabolites. (**C**) Venn diagram demonstrates the identified 3624 metabolites from the LC-MS/MS analysis, out of which 109 were annotated with the NIST and MS-DIAL library; setting the significance criteria at *p*-value < 0.05, eight significant metabolites were obtained, with four metabolites upregulated by the fold change >1.5, one metabolite downregulated by the fold change <0.8, and 1-methylnicotinamide was selected for further analysis with an upregulated fold change of 1.62.

**Figure 3:**
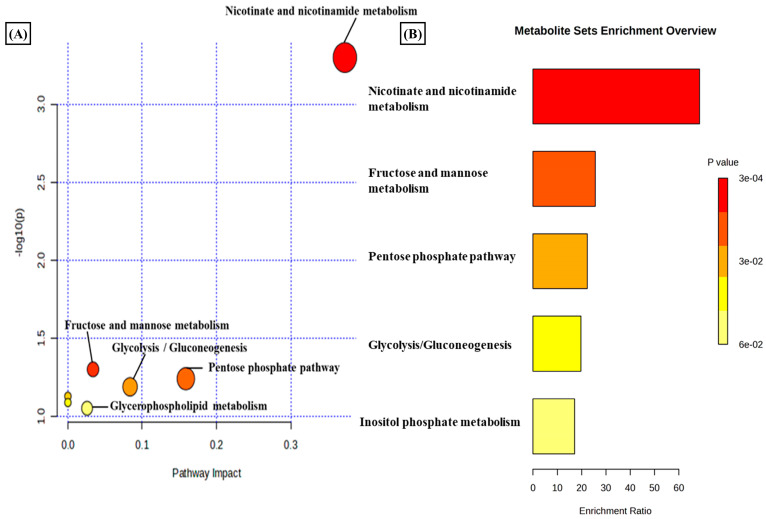

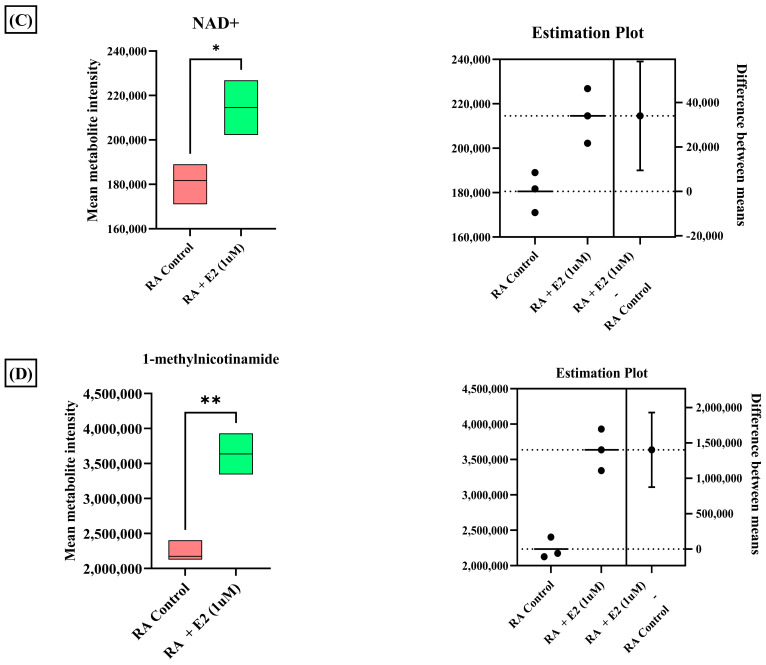
(**A**) KEGG pathway analysis of eight significant metabolites (*p* < 0.05) as analyzed by MetaboAnalyst 6.0. (**B**) Histogram Representation of KEGG enrichment analysis of the eight significant metabolites. Cellular metabolite responses between untreated RA-FLS and E2-treated RA-FLS groups were expressed by mean metabolite intensity calculated by LC-MS analysis. (**C**) Box plot of mean metabolite intensity of NAD^+^. (**D**) Box plot of mean metabolite intensity of 1-methynicotinamide (1-MNA). Error bars represent the standard error of the mean (*n* = 3 each). * *p* < 0.05, ** *p* < 0.01.

A total of 3624 metabolic features were screened against the NIST library and MS-Dial tool to annotate them to known metabolites, which unveiled 109 annotated features. MetaboAnalyst 6.0 was utilized for pathway analysis and enrichment analysis of 109 annotated metabolites from the KEGG pathway library. The pathway analysis revealed seven significant metabolic pathways related to annotated metabolic features (Figure 2A). The metabolic pathways were metabolism related to phenylalanine, tyrosine, tryptophan, valine, leucine, and isoleucine biosynthesis, nicotinate and nicotinamide metabolism, and glutathione metabolism represented by their significance value (*p*-value < 0.05) and the number of metabolites that are associated with these pathways (Table S3). Further, the enrichment analysis also indicated the most altered metabolites and their related pathways with increasing statistical significance (Figure 2B). Additionally, we found eight metabolites with a significant threshold *p*-value set at <0.05 among the 109 annotated metabolites (Table S4). Among the eight metabolites, four metabolites (1-methylnicotinamide, Glyceraldehyde, 3-dihydrogen phosphate, lactobionic acid, Trp-Thr, and 1-hydroxy-10-methylacridone) were upregulated with the fold change threshold set at >1.5, and one metabolite (LPC 14:0) was downregulated with the fold change threshold set at <0.8 (Figure 2C). The pathway analysis and enrichment analysis of eight significant metabolites were also performed with MetaboAnalyst 6.0 (Figure 3A,B), and both the analyses indicated that nicotinate and nicotinamide metabolism was most significantly altered by the impact of E2 on RA-FLS amongst all the pathways affected (Table S5) due to higher overlap with the set of metabolites analyzed. We analyzed the two significant metabolites associated with this pathway. Both the metabolites, NAD^+^ (*p*-value = 0.0183) and 1-methynicotinamide (*p*-value = 0.0018), were upregulated by E2 treatment in RA-FLS, as shown by the box plot in Figure 3C,D.

### 3.3. Estradiol Affects RA Pathogenesis by Stimulating 1-Methyl Nicotinamide

**Figure 4:**
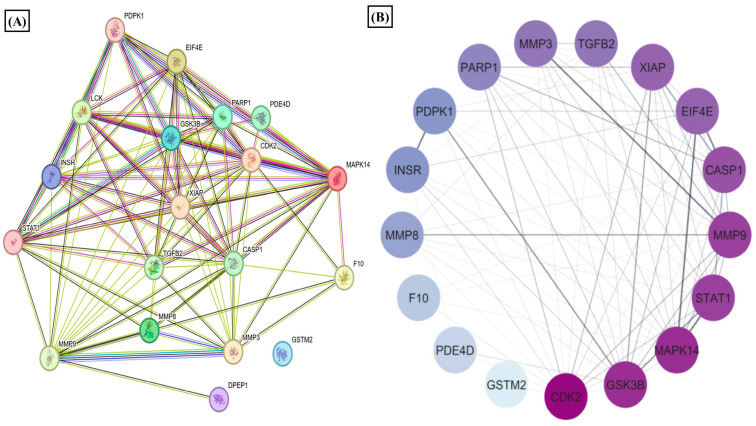

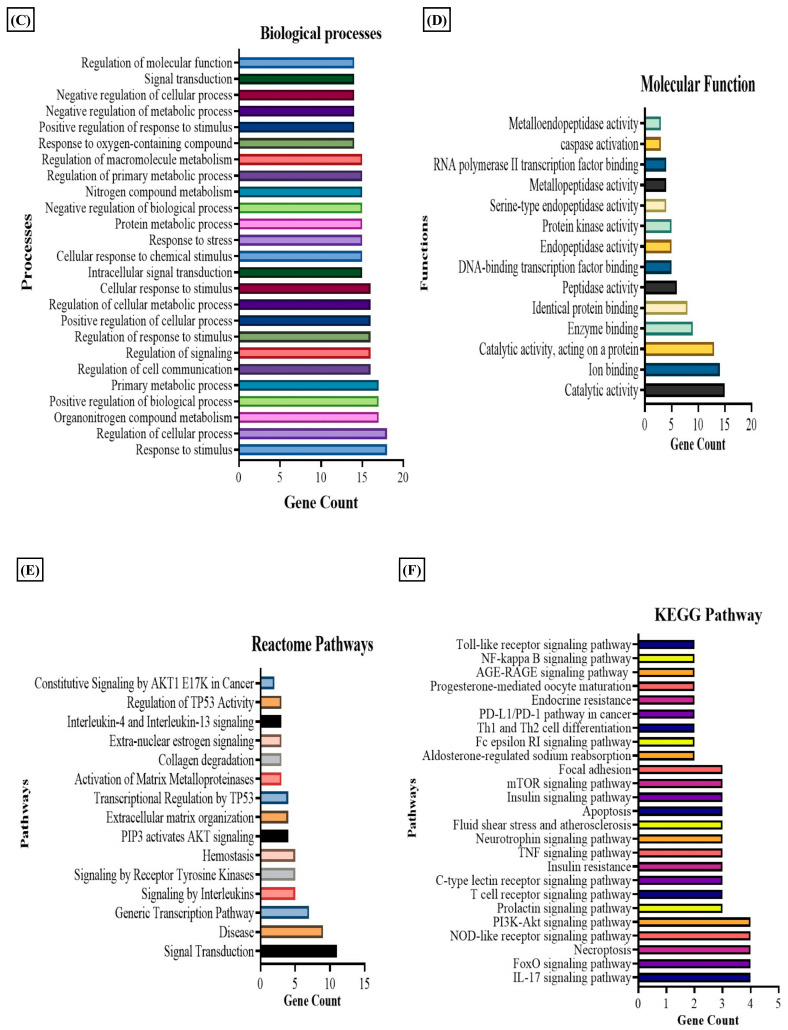
Results of PPI, KEGG, Reactome, and GO analysis of 19 common proteins that were targets of 1-MNA and RA-associated gene targets. (**A**) The PPI network of 19 common proteins was obtained from STRING analysis. (**B**) PPI network of 19 common proteins as constructed by Cytoscape, based on the degree of contribution, where the color of the node represents the degree of contribution of that node in the network. Histogram representation of (**C**) biological process, (**D**) molecular function, (**E**) KEGG pathways, and (**F**) Reactome pathways associated with the 19 common proteins.

We observed that nicotinate and nicotinamide metabolism was modulated by the E2 treatment in RA. Thereby, we further evaluated the effect of this metabolic pathway on RA through E2 induction. The report shows that 1-methyl nicotinamide (1-MNA) is a central metabolite that is synthesized by N-methylation of nicotinamide [50], known to show a significant reduction in RA plasma [51]. Our data depicted its significant upregulation, implying that E2 stimulates nicotinamide biosynthesis. The downstream analysis of the potential gene targets of 1-MNA was therefore obtained with the help of the PharmMapper database. The 1-MNA gene targets (Table S7) were then matched with gene targets of RA retrieved from the DisGeNET database. The comparison found 19 proteins that were potential targets of 1-MNA, and are also related to RA pathogenesis (Table S6). The PPI of these common 19 proteins was constructed with the STRING software (Figure 4A), and the degree of interaction of proteins was analyzed with Cytoscape (Figure 4B) to predict the functional association between the protein that contributes to specific molecular functions or biological processes. MAPK14, MMP9, MMP3, and STAT1 were found to be highly correlated with RA and were also potential targets of 1-MNA. The 19 proteins were also examined using GO and KEGG pathway analysis. The results of the GO analysis revealed 200 entries, out of which 186 belong to biological processes (Table S8) and 14 to molecular functions (Table S9) that were associated with these proteins. This illustrates the response to stimulus, stress, and regulation of the cellular process, cell communication, and signaling as the major biological processes regulated by these proteins (Figure 4C). Catalytic activity, ion binding, enzyme binding, and peptide activity were the essential molecular functions that were found to be related to these proteins (Figure 4D). The KEGG and Reactome pathway analysis (Tables S10 and S11) depicted IL-17 signaling, T cell receptor signaling, TNF signaling, and apoptosis as the predominant KEGG pathways (Figure 4E). Signaling by interleukins, AKT signaling, and collagen degradation as the vital Reactome pathways (Figure 4F) were linked with these 19 proteins.

### 3.4. Estradiol Alters STAT1 Signaling in RA Synovial Fibroblast through 1-Methylnicotinamide

**Figure 5:**
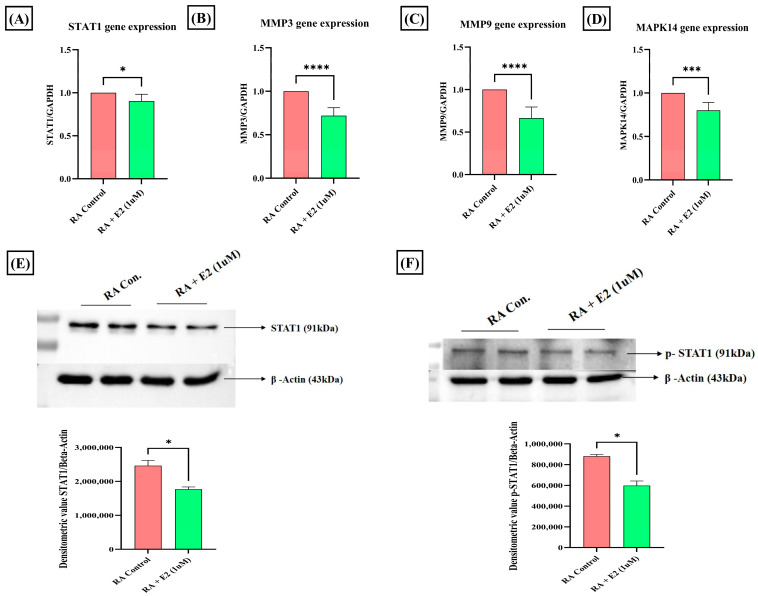
Effect of 1 uM E2 induction for 24 h in RA-FLS on STAT1, p-STAT1, MMPs, and MAPK14 as analyzed by RT-PCR and Western blot analysis compared to untreated RA-FLS as control. (**A**,**E**) Relative gene and protein expression of STAT1. (**B**,**C**) Relative gene expression of MMP3 and MMP9. (**D**) Relative gene expression of MAPK14. (**F**) Relative protein expression of the phosphorylated form of STAT1 (p-STAT1). GAPDH was used as internal control for RT-PCR. Beta-actin was used as loading control for Western blotting. Data are presented as the mean ± standard deviation, * *p* < 0.05, *** *p* < 0.001, **** *p* < 0.0001.

Among the common nineteen proteins analyzed by GO and KEGG enrichment analysis, four proteins—STAT1, MAPK14, MMP3, and MMP9—were selected for further evaluation based on their Gene–Disease Association (GDA) score (Table S6) obtained from the DisGeNET database. STAT1 was the foremost target, with the highest GDA score of 0.4. Upregulated expression of STAT1 protein and its phosphorylated form has been studied in RA synoviocytes [52], and its activation in the cell, primarily stimulated by different pro-inflammatory cytokines (IFNγ, IL-2, IL-6, IL-7, and IL-21), has been reported to promote cartilage degradation [53]. To evaluate the effect of E2 treatment on STAT1 activation, we analyzed its protein and mRNA expression and found that E2 treatment significantly downregulated the STAT1 expression compared to the control (Figure 5A,E). Activation of STAT1 essentially requires its phosphorylation [54]. Thus, we also examined the effect of E2 on the expression of phosphorylated STAT1 and it was found that E2 also decreases the expression of phospho-STAT1 compared to the control (Figure 5F). The results imply that the activities of E2 induction in modulating STAT1 signaling in RA could be promoted by the upregulation of 1-MNA. 

### 3.5. Estradiol Downregulates Matrix-Degrading Enzymes and MAPK14 Expression in RA-FLS Mediated by 1-Methylnicotinamide

The expression of MMP3, MMP9, and MAPK14 was also analyzed after E2 treatment. Matrix metalloproteinases (MMPs) essentially involved in cartilage degradation have increased serum concentration in RA patients and were found to be positively correlated with the disease activity [55,56]. Our results depicted that E2 significantly downregulates the mRNA expression of these matrix-degrading enzymes, MMP3 and MMP9 (Figure 5B,C). MAPK14, a serine-threonine kinase, has been widely assessed for its role in promoting tumorigenesis [57], and its deletion in mice improves the clinical symptoms of TNF-induced arthritis [58]. The mRNA expression analysis of MAPK14 demonstrates that E2 significantly decreases its expression compared to the control group in RA-FLS (Figure 5D). The findings thus showed that the downregulation of MMPs and MAPK14 could be attributed to 1-MNA upregulation in E2-treated RA-FLS. 

### 3.6. Estradiol Ameliorates ROS Production in RA-FLS Regulated by 1-MNA Generation

**Figure 6:**
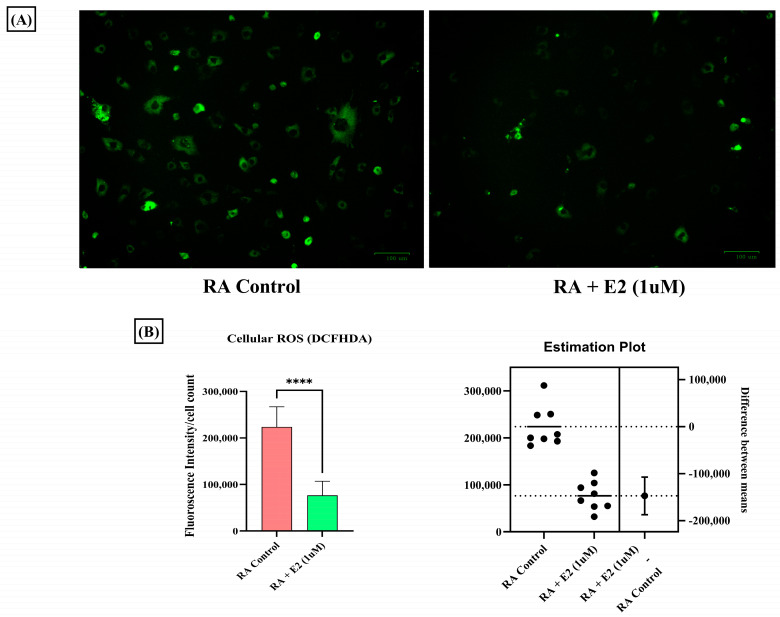
Intracellular ROS level measured by DCFHDA probe in RA-FLS control and pre-treated RA-FLS with 1 uM E2 for 24 h depicted a significant decrease in ROS production after E2 induction. (**A**) Representative fluorescent images of the analysis; the scale bar represents 100 µM. (**B**) Relative fluorescence intensity normalized with cell count. Results are presented as the mean ± standard derivations, **** *p* < 0.0001.

1-MNA has been studied to decrease cellular ROS production in a dose-dependent manner [59], and abundant oxidative stress exhibited by increased ROS generation in RA patients has been demonstrated [60]. Therefore, we also studied the effect of E2 on ROS generation in RA-FLS and observed the decreased DCFDA fluorescence intensity in RA-FLS after E2 exposure (Figure 6A,B). Our findings indicate that E2 inhibits the increased oxidative stress in RA-FLS, which could be attributed to the increased production of 1-MNA metabolite concentration in RA-FLS.

## 4. Discussion

Rheumatoid arthritis, as an autoimmune inflammatory disorder, involves consecutive metabolic alterations to support changes in the synovial microenvironment, such as migration, proliferation, and infiltration of immune cells [61]. Studies have identified distinctive metabolic patterns of RA associated with various metabolic pathways, such as the tricarboxylic acid (TCA) cycle, fatty acids, and amino acid metabolism [29]. A therapeutic strategy to target these metabolic alterations has been considered for RA remission. However, identifying metabolic signatures in serum, plasma, and urine provides limited knowledge about joint metabolism [62]. The altered phenotype of synovial fibroblasts in the synovial joint drives the prominent inflammatory degradation in RA [63]. As the primary effectors, cells regulating joint damage targeting change in the cellular metabolism of RA-FLS offer a promising therapeutic strategy in RA [64]. Recent studies have been conducted to map the dynamic metabolic alteration that occurs in RA-FLS, and a differential metabolic profile has been generated compared to FLS isolated from patients with other inflammatory diseases [65]. The different metabolic pathways are involved in the activation of RA-FLS, and studying the alteration in the metabolites involved in these pathways can provide insight into the development of RA-FLS-directed therapeutic strategies [66]. Therefore, in our study, we applied cellular metabolomics using the LC-MS/MS approach to study the metabolic changes in the synovial fibroblasts of the synovial joint. In addition to inflammatory conditions and autoimmunity development, the hormonal response also plays an essential role in the progression of RA, and the information comes from the fact that RA has been found to be predominant in women [67]. Estrogen has been widely considered to be associated with clinical features of RA [68], and studies have also identified its therapeutic potential in the pathogenesis of RA [69]. Since estrogen closely regulates the metabolism in RA, we studied the effect of estrogen on the metabolic alteration that occurs in synovial fibroblasts during RA inflammatory progression. Our results revealed that estrogen induced in its potent estradiol form significantly impacts the differential metabolome of RA synovial fibroblasts, and consequently, we identified 3624 *m*/*z* features, out of which 176 were found to be significant (*p*-value < 0.05) (Figure 1A). Based on statistical analysis and annotation of identified metabolic signatures, we demonstrated eight significant metabolites with differential expressions under estrogen’s impact on RA-FLS (Table S3). Using pathway and enrichment analysis, we found that nicotinate and nicotinamide (NAD^+^) metabolism (Figure 3A,B) was highly impacted by E2l treatment on RA-FLS, which depicts its close regulation with RA pathogenesis. Boosting NAD^+^ metabolism has been identified as an anti-inflammatory strategy in treating RA patients [70]. In our analysis, we discovered that estradiol treatment in RA-FLS increased the concentration of NAD^+^ and its associated metabolite, 1-MNA (Figure 3C,D), showing that therapeutic horizons of estrogen extend to the regulation of positive metabolic alterations in RA. 

1-MNA is the primary metabolite of nicotinamide, and its therapeutical efficiency has been discovered in terms of anti-inflammatory and anti-thrombotic activities [71]. With its anti-inflammatory properties, 1-MNA has been considered for use to treat a variety of diseases, and, corresponding to the use of nicotinamide, it possesses no side effects [72,73]. We utilized a computational approach for target identification of the compounds, which helped us to study the compound from a drug-design perspective. Using PharmMapper analysis [74], gene targets associated with RA yielded 19 common proteins (Table S5). These 19 common proteins were analyzed for GO enrichment and KEGG pathway analysis (Figure 4). The pathway analysis found IL-17 signaling, T cell receptor signaling, TNF signaling, and apoptosis as the major pathways regulated by these proteins associated with RA’s prominent clinical features [75]. Among the common nineteen proteins, four 4 top targets (STAT1 (0.4), MMP3 (0.1), MMP9 (0.1), and MAPK14 (0.1)) based on GDA score were selected for further downstream validation. We found that estrogen treatment downregulates the protein and mRNA expression of STAT1 (highest GDA score). STAT1 functions as a transcription factor whose activation is induced by circulating cytokines such as IFN-γ [76]. STAT1 activation in RA synovial fibroblasts promotes inflammatory phenotypes [77]. Our study depicted that STAT1 expression is downregulated by estradiol action (Figure 5A,E), which may be attributed to the upregulation of the 1-MNA axis, as the anti-inflammatory response of 1-MNA has been well explored [78]. Phosphorylation of STAT1 is prominent in its activation, and increased phosphorylation has been reported in RA [79,80]. Thus, we also analyzed the protein expression of pSTAT1 on E2 treatment, and it was shown that it significantly downregulated the phosphorylation of STAT1 in RA synovial fibroblasts (Figure 5F).

The other proteins (MMP3 and MMP9) are the matrix metalloproteinases (MMPs), which are mainly proteases and physiologically regulate and modulate the degradation of extracellular matrix (ECM) proteins, primarily collagen and fibronectin [81]. Bone and cartilage degradation are the prominent features of RA and MMPs, mainly produced by synovial fibroblasts to facilitate cartilage degradation in affected synovial joints [82]. Hence, the mRNA expression of these prominent proteins (MMP3 and MMP9) was also analyzed upon estrogen exposure in RA synovial fibroblasts, and it was found that E2 successfully downregulates the expression of MMP3 and MMP9; this aids in the remission of cartilage degradation, which peaks in RA (Figure 5B,C). In our study, these MMPs (MMP3 and MMP 9) were also found to be direct gene targets of 1-MNA; therefore, it may be concluded that estrogen downregulates the expression mediated by 1-MNA. Another common protein, MAPK14/p38α, is a pivotal protein involved in inflammatory and cardiac conditions, as its activation causes the release of prominent pro-inflammatory cytokines [83]. In RA, the predominant expression of MAPK14 was observed in the inflamed tissue of the RA patient, depicting its dominant contribution to chronic inflammation [84]. MAPK14 mRNA expression was therefore analyzed by estrogenic induction. We found that E2 targets the downregulation of the expression of MAPK14 in RA synovial fibroblasts, which can be attributed to 1-MNA metabolic upregulation (Figure 5D). 

Oxidative stress is a significant facet in the pathogenesis of RA that manifests in the form of increased ROS concentration [85]. Increased oxidative stress in RA potentiates the mechanism of MMPs [56], STAT1 [86], and MAPK [87] pathway activation. Analyses of the increased oxidative stress in RA synovial fibroblasts and of the impact of E2 through cellular ROS estimation by DCFHDA analysis were therefore conducted, and revealed that estrogen significantly decreases ROS production in RA synovial fibroblasts, as estimated by fluorescence intensity of DCFHDA (Figure 6A,B). Reduced oxidative stress by estrogen action is also associated with 1-MNA, as studies have identified that the metabolite ameliorates oxidative stress [88]. Projecting 1-MNA as a therapeutic agent through estrogenic action in joint metabolism of RA holds immense potential; however, validation requires a large sample size with a targeted approach. 

## 5. Conclusions

In conclusion, the findings of this study underscore the significant role of estrogen in modulating the concentration of various metabolites in RA-synovial fibroblasts. By applying an LC/MS-MS-based metabolomics approach, we observed that estradiol administration led to a notable increase in metabolites such as NAD+ and 1-MNA, which were associated with nicotinate and nicotinamide metabolism, amongst which 1-MNA was selected as a focal point for further investigation. For a drug targeting approach, we undertook subsequent PharmMapper analysis, and it was revealed that 1-MNA targets essential genes such as STAT1, MAPK14, MMP9, and MMP3, which are involved in the progression of RA. Estradiol significantly downregulated the expression of these genes, potentially mediated by the elevated levels of 1-MNA. Furthermore, the observed reduction in ROS production in RA-synovial fibroblasts upon estrogenic impact suggests its pivotal role in mitigating oxidative stress, a hallmark of the disease. These findings suggest that estrogen may serve as a protective agent against inflammatory progression in RA by modulating the expression of inflammatory genes and mitigating oxidative stress, with the increased concentration of 1-MNA likely playing a central mechanistic role.

## Data Availability

All data that support the findings of the study are within the manuscript or in Supplemental Materials.

## Supplementary Materials

The following supporting information can be downloaded at: https://www.mdpi.com/article/10.3390/metabo14040214/s1, Table S1: The clinical demography characteristics of patients with RA; Table S2: Primer name and sequence; Table S3: The significant metabolic pathways of 109 annotated metabolites from KEGG pathway library analyzed by MetaboAnalyst software; Table S4: The eight significant metabolites based on the *p*-value detailing the *m*/*z* value, fold change and HDMB ID; Table S5: The significant metabolic pathways of eight significant metabolites from the KEGG pathway library analyzed by MetaboAnalyst software; Table S6: The matched common proteins obtained from PharmMapper targets of metabolite 1-methylnicotinamide and RA associated gene targets with their GDA score (in Supplementary word file). Table S7: The gene targets associated with 1-methylnicoyinamide (1-MNA) obtained from PharmMapper database; Table S8: The Gene Ontology (GO) analysis depicting the 186 entries of biological processes associated with the obtained 19 common gene targets of 1-methylynicotinamide and rheumatoid arthritis pathogenesis; Table S9: The Gene Ontology (GO) analysis depicting the 14 entries of molecular functions associated with the obtained 19 common gene targets of 1-methylynicotinamide and rheumatoid arthritis pathogenesis; Table S10: The KEGG pathways associated with 19 common gene targets of 1-methylynicotinamide and rheumatoid arthritis pathogenesis; Table S11: The Reactome pathways associated with 19 common gene targets of 1-methylynicotinamide and rheumatoid arthritis pathogenesis (in Supplementary Excel file).
