# Supplementary material for: Unveiling the Nexus: Cellular Metabolomics Unravels the Impact of Estrogen on Nicotinamide Metabolism in Mitigating Rheumatoid Arthritis Pathogenesis
Source: Metabolites. 2024 Apr 11;14(4):214. doi: 10.3390/metabo14040214 (PMC11052502; doi:10.3390/metabo14040214)
Supplement: Supplementary file 1 [file metabolites-14-00214-s001.zip › SUPPLEMENTARY FILE.pdf]

SUPPLEMENTARY FILE

Unveiling the Nexus: Cellular Metabolomics Unravels the Impact of Estrogen on Nicotinamide Metabolism in Mitigating Rheumatoid Arthritis Pathogenesis

Swati Malik [1,2], Debolina Chakraborty [1,2], Prachi Agnihotri [1,2], Vijay Kumar [3] and Sagarika Biswas [1,2,*]

[1] Department of Integrative and Functional Biology, CSIR- Institute of Genomics and Integrative Biology, Mall Road, Delhi 110007, India; swati.malik@igib.res.in (S.M.); debolina@igib.res.in (D.C.); prachi.igib22a@acsir.res.in (P.A.)

[2] AcSIR- Academy of Scientific and Innovative Research, Ghaziabad 201002, India

[3] Department of Orthopaedics, AIIMS- All India Institute of Medical Sciences, Ansari Nagar, New Delhi 110029, India; vkgene@aiims.edu

* Correspondence: sagarika.biswas@igib.res.in or sagarika.zollu@gmail.com; Tel.: +91-11-27662581

Table S1. The clinical demography characteristics of patients with RA

**S. No.**	**Patient's characteristics**	**RA patient (n=6)**
1	Age (yrs.)	55± 10
2	Sex (Female, Male)	F
3	ESR (mm/hr)	35 ± 5
4	RF (+ve/-ve)	+ve
5	CRP (mg/L)	80±15
6	Tender Joint	20±6
7	Swollen joints	10±4
8	DAS-28 score	6 ± 0.5
9	Disease duration yrs.	15 ± 5
10	Medication (Yes/No)	Yes

*RA: Rheumatoid arthritis; ESR: Erythrocyte sedimentation rate; RF: Rheumatoid factor; CRP: C-reactive protein; DAS-28: Disease Activity Score-28. The values are expressed as Mean ±SD

Table S2: Primer Name and Sequence

S.no.	Primer	Forward	Reverse
1.	STAT1	5' TGACTTCCATGCGGTTGAAC 3'	5' TCCTTTGGCCTGGAGTAATACT 3'
2.	MMP3	5' CACTGGGATAGGAGGGGATG 3'	5' TCTGTGGGTGCCATTTCTGT 3'
3.	MMP9	5' TGCGTGGAGAGTCGAAATCT 3'	5' GGTGATGTTGTGGTGGTGC
4.	MAPK14	5' CCCGAGCGTTACCAGAACC 3'	5' TCGCATGAATGATGGACTGAAT 3'

Table S3: The significant metabolic pathways of 109 annotated metabolites from KEGG pathway library analysed by MetaboAnalyst software

S.no.	Pathway Name	p-value
1.	Phenylalanine, tyrosine and tryptophan biosynthesis	0.001
2.	Valine, leucine and isoleucine biosynthesis	0.00452
3.	Phenylalanine metabolism	0.00452
4.	Glutathione metabolism	0.0054
5.	Arginine biosynthesis	0.014
6.	Nicotinate and nicotinamide metabolism	0.01602
7.	Pantothenate and CoA biosynthesis	0.02785

Table S4: The 8 significant metabolites based on the p-value detailing the m/z value, Fold change and HDMB ID

S.no.	m/z	HMDB ID	Metabolite Name	Fold change	p-value
1.	512.3068	HMDB0010379	LPC 14:0	0.689485	0.000623
2.	137.0705	HMDB0000699	1-Methylnicotinamide	1.627562	0.00547
3.	169.0633	HMDB0001112	Glyceraldehyde, 3-(dihydrogen phosphate)	1.678601	0.00728
4.	277.0928	HMDB0030791	Dihydromethysticin	1.419794	0.00763
5.	381.2486	HMDB0253945	Lactobionic acid	1.970484	0.011718
6.	664.1139	HMDB0000902	beta-Nicotinamide adenine dinucleotide	1.188219	0.021411
7.	305.9473	HMDB0029093	Trp-Thr	1.67495	0.031722
8.	248.0668	HMDB0032983	1-hydroxy-10-methylacridone	1.736537	0.049748

Table S5: The significant metabolic pathways of 8 significant metabolites from KEGG pathway library analysed by MetaboAnalyst software

S.no.	Pathway Name	p-Value
1.	Nicotinate and nicotinamide metabolism	2.53E-04
2.	Fructose and mannose metabolism	0.037637
3.	Pentose phosphate pathway	0.0432
4.	Glycolysis / Gluconeogenesis	0.048741
5.	Inositol phosphate metabolism	0.056096

Table S6: The matched common proteins obtained from Pharmmapper targets of metabolite 1-methylnicotinamide and RA associated gene targets with their GDA score

S.no.	Gene	Gene ID	UniProt	Gene Full Name	GDA Score
1	STAT1	6772	P42224	signal transducer and activator of transcription 1	0.4
2	MAPK14	1432	Q16539	mitogen-activated protein kinase 14	0.1
3	MMP3	4314	P08254	matrix metallopeptidase 3	0.1
4	MMP9	4318	P14780	matrix metallopeptidase 9	0.1
5	PARP1	142	P09874	Poly (ADP-ribose) polymerase 1	0.09
6	MMP8	4317	P22894	matrix metallopeptidase 8	0.08
7	CASP1	834	P29466	caspase 1	0.07
8	GSTM2	2946	P28161	glutathione S-transferase mu 2	0.05
9	DPEP1	1800	P16444	dipeptidase 1	0.03
10	CDK2	1017	P24941	cyclin dependent kinase 2	0.02
11	GSK3B	2932	P49841	glycogen synthase kinase 3 beta	0.02
12	INSR	3643	P06213	insulin receptor	0.02
13	PDPK1	5170	O15530	3-phosphoinositide dependent protein kinase 1	0.01
14	PDE4D	5144	Q08499	phosphodiesterase 4D	0.01
15	LCK	3932	P06239	LCK proto-oncogene, Src family tyrosine kinase	0.01
16	TGFB2	7042	P61812	transforming growth factor beta 2	0.01
17	XIAP	331	P98170	X-linked inhibitor of apoptosis	0.01
18	F10	2159	P00742	coagulation factor X	0.01
19	EIF4E	1977	P06730	eukaryotic translation initiation factor 4E	0.01

Table S7: The gene targets associated with 1-methylnicoyinamide (1-MNA) obtained from Pharmmapper database (**In excel file**)

Table S8: The Gene Ontology (GO) analysis depicting the 186 entries of Biological process associated with obtained 19 common gene targets of 1-methylynicotinamide and Rheumatoid arthritis pathogenesis (**In excel file**)

Table S9: The Gene Ontology (GO) analysis depicting the 14 entries of Molecular functions associated with obtained 19 common gene targets of 1-methylynicotinamide and Rheumatoid arthritis pathogenesis (**In excel file**)

Table S10: The KEGG pathways associated with 19 common gene targets of 1-methylynicotinamide and Rheumatoid arthritis pathogenesis (**In excel file**)

Tabel S11: The Reactome pathways associated with 19 common gene targets of 1-methylynicotinamide and Rheumatoid arthritis pathogenesis (**In excel file**)
